# Feasibility of Two Educational Methods for Teaching New Mothers: A Pilot Study

**DOI:** 10.2196/ijmr.4583

**Published:** 2015-10-08

**Authors:** M Cynthia Logsdon, Deborah Davis, Diane Eckert, Frances Smith, Reetta Stikes, Jeff Rushton, John Myers, Joshua Capps, Kathryn Sparks

**Affiliations:** ^1^ University of Louisville School of Nursing Louisville, KY United States; ^2^ University of Louisville Hospital Louisville, KY United States; ^3^ University of Louisville Department of Pediatrics Louisville, KY United States; ^4^ Center for Women and Infants University of Louisville Hospital Louisville, KY United States; ^5^ University of Louisville Louisville, KY United States

**Keywords:** Patient Education as Topic, Video Recording, Postpartum Period, Utilization, Nurses, Personal Satisfaction

## Abstract

**Background:**

Printed health educational materials are commonly issued to prepare patients for hospital discharge. Teaching methods that engage multiple senses have been shown to positively affect learning outcomes, suggesting that paper materials may not be the most effective approach when educating new mothers. In addition, many written patient educational materials do not meet national health literacy guidelines. Videos that stimulate visual and auditory senses provide an alternative, potentially more effective, strategy for delivering health information. The acceptability of these methods, as perceived by nurses executing patient education initiatives, is important for determining the most appropriate strategy.

**Objective:**

The purpose of this study was to determine the feasibility of 2 educational methods for teaching new mothers how to care for themselves and their infants after hospital discharge. Feasibility was measured by adequate enrollment, acceptability of the intervention to patients and nurses, and initial efficacy.

**Methods:**

New mothers (n=98) on a Mother-Baby Unit received health information focused on self-care and infant care delivered as either simple printed materials or YouTube videos on an iPad. Mothers completed a pretest, post-test, and an acceptability survey. Following completion of the initiative, nurses who participated in delivering the health education using one of these 2 methods were asked to complete a survey to determine their satisfaction with and confidence in using the materials.

**Results:**

Mothers, on average, were 26 years old; 72% had a high school education; and 41% were African American. The improvement in knowledge scores was significantly higher for the iPad group (8.6% vs 4.4%, *P*=.02) compared to the pamphlet group. Group (*B*=4.81, *P*=.36) and time (*B*=6.12, *P*<.001) significantly affected scores, while no significant interaction effect was observed (*B*=5.69, *P*=.09). There were no significant differences in responses between the groups (all *P* values >.05). The nurses had a mean age of 44.3 years (SD 13.9) and had, on average, 16.6 years of experience (SD 13.8). The nurses felt confident and satisfied administering both educational modalities.

**Conclusions:**

The pamphlet and iPad were identified as feasible and acceptable modalities for educating new mothers about self-care and infant care, though the iPad was more effective in improving knowledge. Understanding the acceptability of different teaching methods to patient educators is important for successful delivery of informational materials at discharge.

## Introduction

### Background

International health care providers have long acknowledged the importance of sharing health information with patients/consumers. In no population group is education more important than childbearing women and their families [1]. The World Health Organization recommends that essential health content be taught to pregnant and parenting women to protect their health and that of their babies (eg, postnatal recovery, care of the newborn, promotion of early exclusive breastfeeding, and assistance with deciding on future pregnancies to improve pregnancy outcomes) [1]. However, childbearing women frequently have difficulty interpreting and operationalizing information, and health education may not translate into appropriate health behaviors [2].

In the United States, emphasis on health education for all patient groups is unprecedented. The Affordable Care Act encourages patients to take control of their health care decisions based upon the latest evidence [3]. In the acute care setting, national organizations such as the Agency for Healthcare Research and Quality, American Medical Association, Centers for Medicare and Medicaid Services, and Patient-Centered Outcomes Research Institute stress the need for effective health education for hospitalized patients and an evaluation of health education is included in hospital accreditation procedures [4]. Soon, reimbursement to acute care settings will be based upon such quality measures.

Testing innovative methods for teaching new mothers, with attention to health literacy levels of the population, should be guided by efforts to improve state maternal child health statistics [5-7]. Kentucky has one of the lowest literacy rates in the United States with 14% of adults 16-65 years of age, on average, having very little to no literacy skills and another 26% having low literacy skills [8]. Simultaneously, the rate of substantiated cases of child abuse in Kentucky is 16.6 per 1000 children, compared to the US rate of 9.1 in 2011 [9]. Traumatic brain injury is the leading cause of death for children and 64% of cases are from abuse [10]. The US has a higher rate of fatalities from child abuse and neglect than any other higher income country and Kentucky has ranked among the states with the highest rate over recent years [11]. Kentucky is currently ranked 8th among all states for child abuse fatalities [11]. Breastfeeding statistics from Kentucky are also poorer than the US average for all indicators including rates of ever breastfeeding (United States 77% vs Kentucky 59%), breastfeeding at 6 months (47% vs 27%), breastfeeding at 12 months (26% vs 11%), exclusive breastfeeding at 3 months (36% vs 21%), and exclusive breastfeeding at 6 months (16% vs 10%) [12]. Rates of postpartum depression, which have an adverse impact on development of both the mother and infant, are greater than or equal to national rates in Kentucky. Poverty is a risk factor for all of these and many other threats to women and children’s health [13-19]. These data suggest that innovative methods for teaching new mothers, with attention to health literacy levels of the population, are needed if we are to improve state statistics and address health issues that are frequently associated with poverty in women and children.

In Kentucky, like the remainder of the United States, most women deliver their newborns in hospitals and are discharged from the hospital 2-3 days after birth. In hospitals, most health education has traditionally consisted of providing verbal instruction and written health education materials before hospital discharge, but the efficacy and acceptability of these methods have not been comprehensively evaluated. In 2005, the Center for Medicare and Medicaid Services developed a standardized survey, the Hospital Consumer Assessment of Healthcare and Systems, to measure patients’ perspectives on the quality of hospital stays. The survey is administered by outside companies and includes questions about communication with nurses and discharge education.

Key to effective patient education is tailoring materials and messages to appropriate literacy levels and preferred learning styles of patients [20,21], especially in families who are at risk for adverse outcomes due to low education and/or low literacy levels. Additionally important is teachers’ satisfaction with, and confidence in, using methods and materials when educating new mothers and families [22]. The purpose of this study was to determine the feasibility of 2 educational methods for teaching new mothers how to care for themselves and their infants after hospital discharge. Feasibility was measured by adequate enrollment, acceptability of the intervention to patients and nurses, and initial efficacy as described in a tutorial on pilot studies by Thabane et al [23].

### Review of the Literature

Written educational materials are widely used at hospital discharge but may not be the most effective vehicle for educating today’s generation of new mothers [20,24,25]. Teaching that engages multiple senses has been shown to enhance learning [26,27]. For example, it has long been known that videotapes can portray real-life situations; employ actors, graphics, and words that are appropriate for a particular population; improve short-term knowledge [27]; and enhance retention of information better than written materials [28]. Research on dual coding theory has determined that when individuals both see and hear an explanation, they are able to generate more creative solutions to solve problems [26]. Dual coding theory assumes that there are 2 cognitive subsystems: one processes nonverbal events (imagery) and the other specializes in language. New technology that includes video and engages 2 cognitive subsystems provides an alternative, and potentially more effective, way to deliver health information [29].

Increasingly, pregnant and parenting women are using technology to access health information [30]. In a recent US survey (Listening to Mothers III), nearly two thirds (64%) of pregnant or parenting women accessed online health information from a mobile phone in a typical week and 82% went on the Internet from a computer [31]. Women also reported using tablet devices (35%) and iPod Touch devices (21%) to access information on the Internet. Further testing is needed to determine which technology is most effective and acceptable. While some studies have shown that low-income individuals are less likely to access the Internet, it was concluded that decreasing literacy demands would increase accessibility and use of information [32,33].

New mothers are often overwhelmed with the amount of new information that they are given at hospital discharge. In order to enhance learning, it may be more effective to focus on essential topics that new mothers must know about self and baby care before they visit a health care provider 2-4 days after hospital discharge [34,35]. One essential topic is knowledge of breastfeeding [36].

Data from our maternity unit, the Center for Women and Infants at the University of Louisville Hospital (ULH), indicate there is room for improvement in our patient education. When asked whether nurses explained discharge information in a way that could be understood, 73.1% of mothers answered “Yes,” which was below the national average of 78% [37]. In addition, our earlier research indicated that some of our written health education for new mothers had a reading level that was too high [38] and that new mothers are comfortable using technology to obtain health information [39]. Thus, our nursing staff were motivated to develop and test an intervention to improve patient education, prompting this study.

## Methods

### Study Design

During a specified period, all mothers on the Mother-Baby Unit were randomized to receive standard teaching or a newly developed teaching module as part of a quality improvement initiative, which included an evaluation component. Mothers were then asked if their data could be included in a research study. The study was approved by the site and the Institutional Review Board of the University.

### Sample

Eligibility criteria included English-speaking mothers with live births, whose babies were not in the neonatal intensive care unit and were expected to be discharged with their birth mothers. Table 1 displays data related to demographics of the sample, for which there were no significant differences between the 2 groups (all *P* values >.05). A majority of the analytic sample were non-Hispanic white (n=39/98, 39.8%) or black (n=40/98, 40.8%) with a high school education (n=71/98, 72.4%) and a mean age of 26.2 years. All nurses on the Mother-Baby Unit who completed discharge teaching during the study period were asked to complete the nurse acceptability survey.

**Table 1:** Overall baseline demographics and stratified by educational modality.

Variable		Overall (n=98) n (%) or mean (SD)	Pamphlet (n=51) n (%) or mean (SD)	iPad (n=47) n (%) or mean (SD)	*P* value
Hispanic		5 (5.1)	2 (3.9)	3 (6.4)	.58
**Ethnicity**					
	White	39 (39.8)	22 (43.1)	17 (36.2)	.62
	Black	40 (40.8)	19 (37.3)	21 (44.7)	
	Other	15 (15.3)	9 (17.7)	6 (12.8)	
**Education**					
	<High school	22 (22.4)	10 (19.6)	12 (25.5)	.75
	High school	71 (72.4)	38 (74.5)	33 (70.2)	
	>High school	5 (5.1)	3 (5.9)	2 (4.2)	
Mean age		26.2 (6.1)	27.2 (6.4)	25.2 (5.6)	.10

### Intervention

The study intervention was developed as follows. First, with guidance from the literature including the Baby Friendly Initiative [34-36], nursing staff and nursing leaders created a list of essential topics that new mothers must be taught before hospital discharge. Information was restricted to that needed by new mothers before their first pediatric office visit 2-3 days after discharge. Second, simple patient education brochures were developed on these topics, based upon national health literacy guidelines [40,41]. Third, the digital media services department of the university created short videos of the content and placed them on the YouTube channel. Fourth, nursing staff critiqued the pamphlets and videos. Minor revisions were made based upon this input. One hour of staff training was completed before initiation of the study. Finally, the YouTube channel was accessed through computer/tablets on the unit. Upon hospital discharge, new mothers were given information about how to access the YouTube channel if further clarification was needed.

### Study Measures

Study measures included an investigator-created assessment of knowledge. Acceptability of the interventions was also measured in mothers and nurses.

### Procedures

Using a table of random numbers, new mothers were randomized into the iPad or pamphlet conditions. After consenting to the study, mothers completed a pretest. After the intervention, they completed a post-test and acceptability survey. Nursing staff on the unit delivered the intervention. We examined the feasibility and acceptability of the 2 differing educational modalities in the new mothers by asking 9 questions, which are described in the “Results” section.

Upon completion of the patient intervention, all nurses on the unit who participated in the new discharge teaching were asked to complete a brief survey to determine their level of acceptance regarding using YouTube videos and iPads to educate postpartum patients. The survey examined nurses’ perceived confidence and satisfaction in delivering the educational modalities through 5 questions, which are described in the “Results” section. The survey was distributed to nurses via email. Anonymous surveys were returned in an envelope to the unit and were picked up by the study team. A reminder email was sent twice before data collection was deemed complete.

### Power and Sample Size Justification

For this study, all new mothers (live births) at ULH were considered. Based on our preliminary studies, we anticipated that 10% of all potential participants would not be eligible and/or willing to participate, and that 10% of the eligible/willing participants would be lost to follow-up. Therefore, we recruited 100 mothers (n=51 in the pamphlet group, and n=49 in the iPad group). This was a feasible sample size for the research team to recruit and enroll for the study. Two (2.0%) were lost to follow-up. All analyses were performed on data for the remaining 98 individuals (n=51 in the pamphlet group and n=47 in the iPad group). Power calculations were based on the anticipated total sample size (n=98) and were used for complete analysis. We developed separate mixed-effects general linear models for each of the outcomes. Based on the anticipated sample size, the study had 84% power to detect a 10% main effect of each treatment for each outcome. Therefore*,* the number of participants in each comparison group was more than sufficient.

### Statistical Analysis

To determine the influence of the iPad versus simple pamphlet on knowledge of self-care and infant care, we started with straightforward tests for differences between the 2 groups of individuals. Independent samples *t* tests were used to test for differences among continuous variables, while chi-square Fischer exact tests and Wilcoxon methods (when appropriate) were used to test for differences among categorical variables. To examine outcome knowledge for self-care and infant care, separate mixed-effects general linear models were developed for each outcome. The educational modalities were analyzed as fixed effects, and time (week since randomization) was analyzed as a repeated measures effect. All main effects and two-way interaction effects were investigated for significance from the mixed-effects models that were developed. Data were collected from the YouTube channel to determine the frequency and duration of access after hospital discharge.

## Results

### Improvement in Outcomes Over Time

As seen in Table 2, the iPad group had lower mean outcome scores at baseline (81.7% vs 84.3%, *P*=.27), but the difference in mean scores was not significant. By contrast, the iPad group had higher scores at follow-up (90.3% vs 88.7%, *P*=.43), but still did not reach significance. However, the improvement in scores was significantly higher for the iPad group (8.7% vs 4.4%, *P*=.02) compared to the pamphlet group.

**Table 2:** Baseline scores, T2 scores, and change in scores over time stratified by group.

Variable	Pamphlet Mean (SD)	iPad Mean (SD)	*P* value
Baseline scores	84.3% (11.0%)	81.7% (12.5%)	.27
T2 Scores	88.7% (10.8%)	90.3% (9.9%)	.43
Change over time	4.4% (8.3%)	8.7% (9.3%)	.02

As seen in Table 3, taking a longitudinal approach, group (*B*=4.81, *P*=.36) and time (*B*=6.12, *P*<.001) significantly affected scores over time, while no significant interaction effect was observed (*B*=5.69, *P*=.09).

**Table 3:** General linear model: scores by group, time, and group by time interaction.

Predictor	*B* (95% CI)	*P* value
Group	4.81 (2.7-9.7)	.04
Time	6.12 (3.8-12.2)	<.001
Group-time interaction	5.69 (2.8, 13.0)	.09

### Feasibility and Acceptability

#### Mothers

As seen in Table 4, new mothers found both the pamphlet and iPad to be feasible and acceptable modalities for receiving education about self-care and infant care. There were no significant differences in feasibility and acceptability responses between the 2 groups (all *P* values >.05).

**Table 4:** Feasibility and acceptability measures for participants overall and stratified by group.

Question	Overall (n=98) n (%)	Pamphlet (n=49) n (%)	iPad (n=45) n (%)	*P* value
Easy to read	89 (94.7)	48 (98.0)	41 (95.3)	.14
Good place for me to learn more about depression	88 (93.6)	48 (98.0)	40 (89.0)	.07
Good place for me to learn more about infant care	90 (95.7)	48 (98.0)	42 (93.3)	.27
Good place for me to learn more about building a bond with my baby	85 (90.4)	45 (91.8)	40 (89.0)	.63
Good place for me to learn more about breastfeeding	87 (92.6)	45 (91.8)	42 (93.3)	.78
Know where to call if I need help with my infant	87 (92.6)	47 (95.9)	40 (89.0)	.20
Know what to do if I need help	88 (93.6)	47 (95.9)	41 (95.3)	.34
Recommend	88 (93.6)	48 (98.0)	40 (89.0)	.07
I am more likely to get treatment if I have depression	85 (90.4)	44 (89.8)	41 (95.3)	.83

### Nurses

The nurses felt confident and satisfied using both the iPad and simple pamphlets, as seen in Table 5. The nurses had a mean age of 44.3 years (SD 13.9) and had, on average, 16.6 years of experience (SD 13.8).

**Table 5:** Nurses’ confidence and satisfaction scores for administering the education modalities.

Question	Mean score (SD)^a^
Confidence in having met the new mother’s and family’s need for teaching	5.94 (0.9)
Confidence in your use of the iPad and YouTube videos for teaching	5.06 (1.6)
Confidence in your use of simple pamphlets for teaching	6.00 (0.9)
Satisfaction with simple pamphlets	6.00 (1.1)
Satisfaction with iPad and YouTube	5.00 (1.7)

^a^Scores range from 1 (very low) to 7 (very high).

### Follow-up Visits to YouTube

Mothers who received the iPad intervention were provided information on how to return to the YouTube Channel to view the videos after hospital discharge; 8 of 45 mothers did so. The topics viewed after discharge were the following: breastfeeding (n=1), bottle feeding (n=1), and critical symptoms in mothers (n=6).

## Discussion

### Principal Findings

The pamphlet and iPad were identified as feasible and acceptable modalities for educating new mothers about self-care and infant care. The nurses felt confident and satisfied administering both educational modalities.

### Limitations

Limitations of the study include data collection from one organization, a cross-sectional design, and the use of investigator-developed questions. In addition, only English-speaking mothers and those with an infant being discharged home with them were included in the study. Our next study will address these limitations. In addition, the results may have been impacted by a ceiling effect, as both interventions were evaluated highly.

### Comparison With Prior Work

In agreement with findings of other researchers [42], YouTube served as an effective method for sharing health information in this study. Further research should test the simultaneous use of written and creative video materials by health literacy level [43].

### Conclusions

These findings provide a foundation to determine whether using the preferred teaching method from this study could improve long-term outcomes for women and their infants, and to examine the cost-effectiveness of delivering health information using technology. This study is in line with funding priorities of national organizations; both the Agency for Healthcare Research and Quality and Patient-Centered Outcomes Research Institute have set priorities to reduce health disparities for those most at risk, such as low-income women and children from inner cities. Results from this study hold great promise for improving the uptake of information among new mothers with limited literacy skills, their health status and that of their baby, and their satisfaction with care [33].

## Abbreviations

ULH: University of Louisville Hospital
